# Rhino-orbital mucormycosis in a patient with no susceptibility following P.vivax malaria infection—a case report

**DOI:** 10.1186/s12886-022-02611-8

**Published:** 2022-10-01

**Authors:** Sonali Prasad, Aman Gaur, Anuj Mehta, Nimisha Kaushal

**Affiliations:** 1 Department of Ophthalmology, Vardhman Mahavir Medical College and Safdarjung Hospital, Near AIIMS, Ansari Nagar West, 110029 Delhi, India; 2 New Delhi, India

**Keywords:** Rhino-orbital mucormycosis, P.vivax malaria, Orbital exenteration, Immunocompetent, Case report

## Abstract

**Background:**

Mucormycosis is a potentially lethal, angioinvasive fungal infection caused by the Mucoracea family comprising Mucor, Rhizopus, and Absidia species. It is commonly associated with uncontrolled diabetes mellitus, the use of corticosteroids, immunosuppressive drugs, and Covid-19 infection. The occurrence of mucormycosis in an immunocompetent patient is rare. Also, only a few case reports have been published where patients developed mucormycosis with associated malarial infection.

**Case presentation:**

A young female presented with a 3-weeks history of painful swelling and outward protrusion of the right eye with complete loss of vision. She had a history of P.vivax malaria two weeks before her ocular symptoms. On ocular examination, there was proptosis and total ophthalmoplegia with loss of corneal sensations in the right eye. Hematological examination revealed normocytic normochromic anemia and thrombocytopenia. MRI was suggestive of right-sided pansinusitis and orbital cellulitis with right superior ophthalmic vein thrombosis and bulky cavernous sinus.

Nasal biopsy was negative for fungal culture. An emergency surgical debridement of all the sinuses was done with right orbital exenteration. Histopathology confirmed the diagnosis of mucormycosis and the patient improved post-operatively on systemic antifungals.

**Conclusion:**

Such an association of mucormycosis with malaria infection is rarely reported in the literature and is hypothesized to be a result of immunosuppression caused by malaria. Also, emphasis is laid upon having a high index of suspicion for fungal infection in the setting of pansinusitis even if the risk factors are absent.

We hereby report a case of rhino-orbital mucormycosis following P.vivax malaria in a 20-year-old female with anemia and thrombocytopenia.

## Background

Mucormycosis is a potentially life-threatening condition caused by a group of invasive filamentous fungi of the Mucoraceae family [1]. Commonly reported risk factors associated with mucormycosis include a history of Covid-19, uncontrolled diabetes especially ketoacidosis, hematological malignancies, prolonged use of corticosteroids, treatment with T cell immunosuppressants, recipients of allogeneic stem cell transplant [2, 3]. The occurrence of mucormycosis in an immunocompetent patient is very rare.

The first case of rhinomaxillary mucormycosis following severe falciform malaria was reported by Katherine et al. [4]. A similar case was reported by Wilson et al. [5]. No published data is available regarding the association of P.vivax malaria and mucormycosis.

We hereby report a case of rhino-orbital mucormycosis following P.vivax malaria in a 20-year-old female with anemia and thrombocytopenia.

## Case presentation

A 20-year-old female presented to our hospital with a complaint of right-sided malar pain followed by gradually progressive painful swelling and outward protrusion of the right eye for 3 weeks with complete loss of vision.

There was no history of diabetes, steroid intake, use of immunomodulators/immunosuppressants, hematological malignancy, organ transplantation, and previous Covid-19 infection. RTPCR done on three different occasions was negative for SARS-CoV-2. However, the patient had a history of P. vivax malarial infection (diagnosed on peripheral smear) two weeks prior to developing sinusitis, for which she received three days of artemisinin-based combination therapy and oral primaquine(for 14 days). Symptomatic remission was achieved from the 3^rd^ day of antimalarial treatment. No further tests were conducted to confirm parasite clearance. There was no history of weight loss or loss of appetite. She was afebrile and her vitals were stable at the time of presentation.

**Fig. 1:**
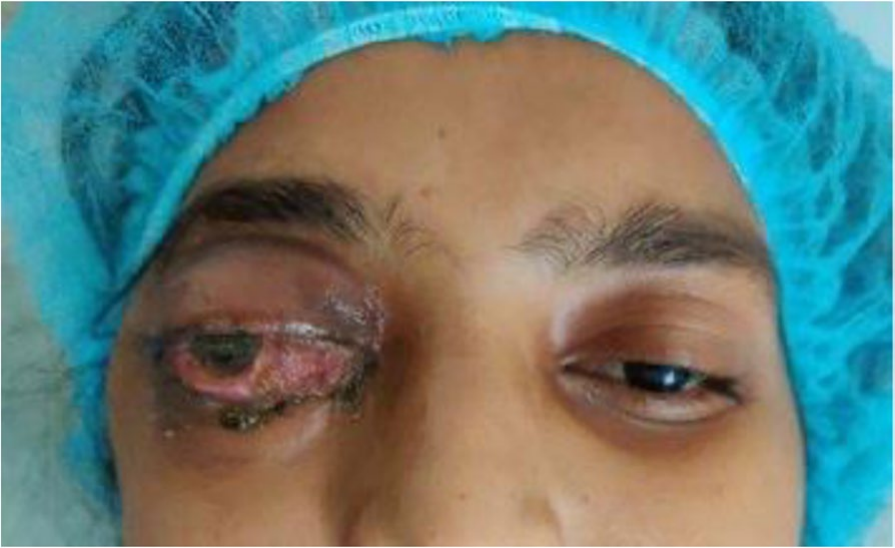
Pre-operative image of the patient showing R/E orbital cellulitis with panophthalmitis

Her vision was no light perception in the right eye and 6/6 in the left eye. On ocular examination, 3 mm of proptosis was noted in the right eye relative to the left eye (on Hertel's exophthalmometry) with total ophthalmoplegia. There was edema of both right upper and lower lids along with ptosis. Conjunctiva was chemosed resulting in lagophthalmos and exposure keratopathy (Fig. 1). Corneal sensations were absent with hypoesthesia in the supra and infraorbital regions. The right eye pupil was 6 mm and nonreactive. Rest anterior chamber and posterior chamber details could not be visualized due to hazy cornea. Examination of the left eye was within normal limits.

## Investigations

Hematological examination reports showed normocytic normochromic anemia (Hb – 8.8 gm%, MCH – 27.9 pg, MCV – 87 fL, MCHC- 32.10 gm/dL), thrombocytopenia (platelet – 73 × 103/mm3), leukocyte count(6500 / mm3), reticulocyte count 1.7%, and differential leukocyte count DLC – N81L16.5E0.1M1.2). Fasting/postprandial blood sugar(98 mg/dl and 145 mg/dl respectively) and HbA1c(4.7%) were within normal limit. D-dimer(480 ng/ml) and coagulation profile(PT/aPTT/INR – 18.50/42.0/1.33) were also normal. Peripheral smear/rapid diagnostic test (HRP2 and pLDH) for the malarial parasite, ELISA for HIV, and blood culture for bacterial/fungal growth were negative. Also, RTPCR of nasal, nasopharyngeal, and oropharyngeal specimens was negative for SARS CoV-2 at the time of presentation.

**Fig. 2:**
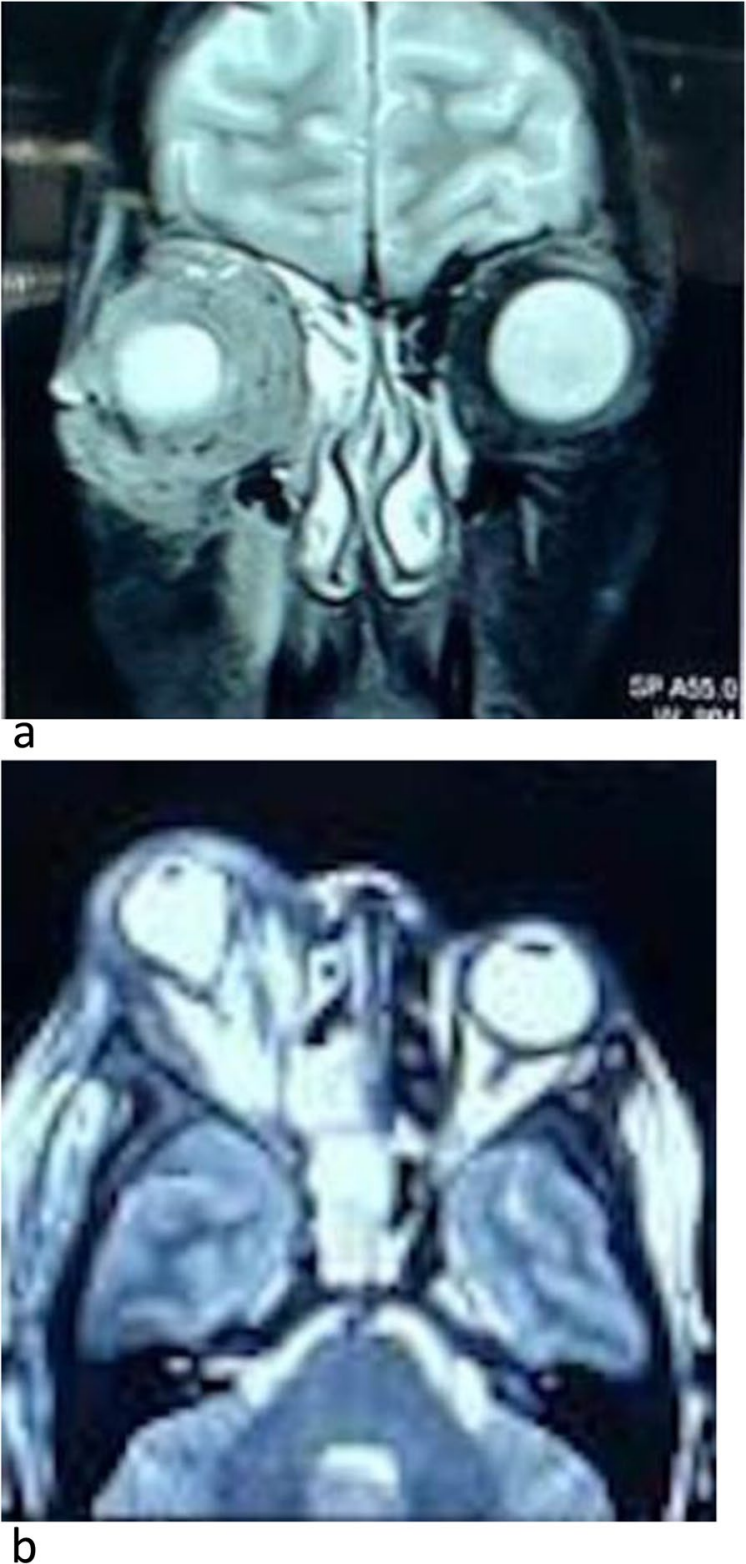
**a**,**b** MRI T2W Flair shows hyperintense soft tissue mucosal thickening with peripheral enhancement seen in right maxillary sinuses, right ethmoid air cell, bilateral sphenoid sinus and right frontal with right orbital and periorbital cellulitis

MRI brain & orbit showed partial cavernous sinus thrombosis, opacification of all sinuses, orbital compartment syndrome along with moderate right-sided axial proptosis with edematous changes in retro-orbital fat (Fig. 2a, b). On MR venography right cavernous sinus appeared bulky with a partial filling defect in post-contrast study representing partial cavernous sinus thrombosis. A partial filling defect in the right superior ophthalmic vein may represent partial occlusion.

Bilateral lung fields were clear on the chest x-ray.

## Differential diagnosis

This is a case of rhino-orbital sinusitis which can be of fungal or bacterial etiology.

## Treatment

Previous records of the patient revealed that she had already received treatment from elsewhere in view of cavernous sinus thrombosis. This included a two-week course of systemic antibiotics (amoxicillin and clavulanic acid) along with anticoagulants (rivaroxaban). However, her condition did not improve.

On our end, she was continued on broad-spectrum intravenous antibiotics (piperacillin and tazobactam) and systemic anti-inflammatory medications (diclofenac sodium and serratiopeptidase). Despite five days of treatment, there was no improvement in clinical condition. Considering non-responsiveness to intensive antibiotic therapy in the setting of pansinusitis with orbital sinusitis, fungal etiology was suspected and oral posaconazole was started from the sixth day of treatment. Nasal biopsy was sent for gram/KOH stain and culture on blood agar/sabourauds dextrose agar which was negative for any bacterial or fungal elements.

As her condition deteriorated further she was planned for orbital exenteration and surgical debridement of paranasal sinuses.

**Fig. 3:**
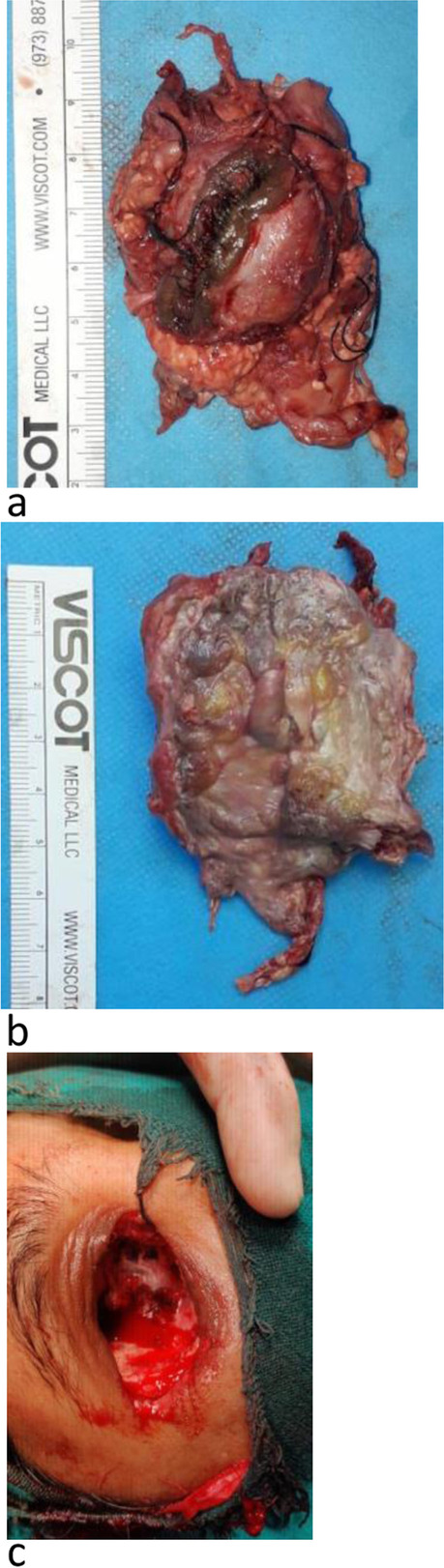
**a** showing exenterated eye with lid margin. **b** showing exenterated eye with optic nerve segment. **c** showing intra-operative empty socket after exenteration of the eye

**Fig. 4:**
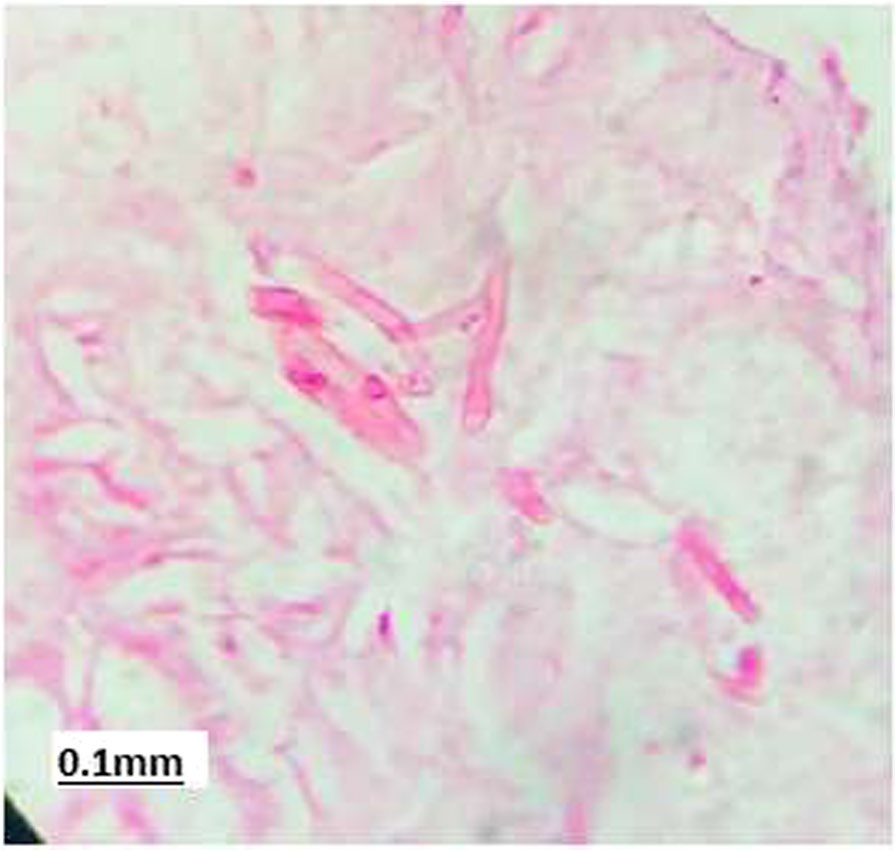
High power light microscopy examination of paraffin embedded section on h & e staining showing broad, aseptate fungal hyphae with right angle branching. Acquisition performed at 40 × magnification corresponding to a pixel resolution of 0.25 mpp. Celleste image analysis software used and downsampling factor equal to 16 was used. The model name of the microscope is Anjay Engitech, brand Anjay, Drawtube metal make, Magnification- 40x- 1000x, light source led, power supply 220 V,Stage size compact. Eye piece-10x & 15 × wide field, objectives 5 × 10x 45x & 100 × wide field. Item code/ batch no- 179,591,590

Right maxillary antrostomy, anterior and posterior ethmoidectomy, and sinusotomy of the sphenoid sinus with septoplasty were performed under general anesthesia followed by lid sparing orbital exenteration of the right eye (Fig. 3a, b, c). Histopathological examination of exenterated orbital tissue measuring 5 × 5x3cm with eyeball and optic nerve revealed inflammation, multinucleated giant cell granulomas, broad ribbon-like aseptate fungal hyphae with right-angle branching confirming mucormycosis. No vascular invasion was noted (Fig. 4).

The patient was started on injection liposomal Amphotericin B 5 mg/kg/day which was given for two weeks.

## Outcome and follow-up

**Fig. 5:**
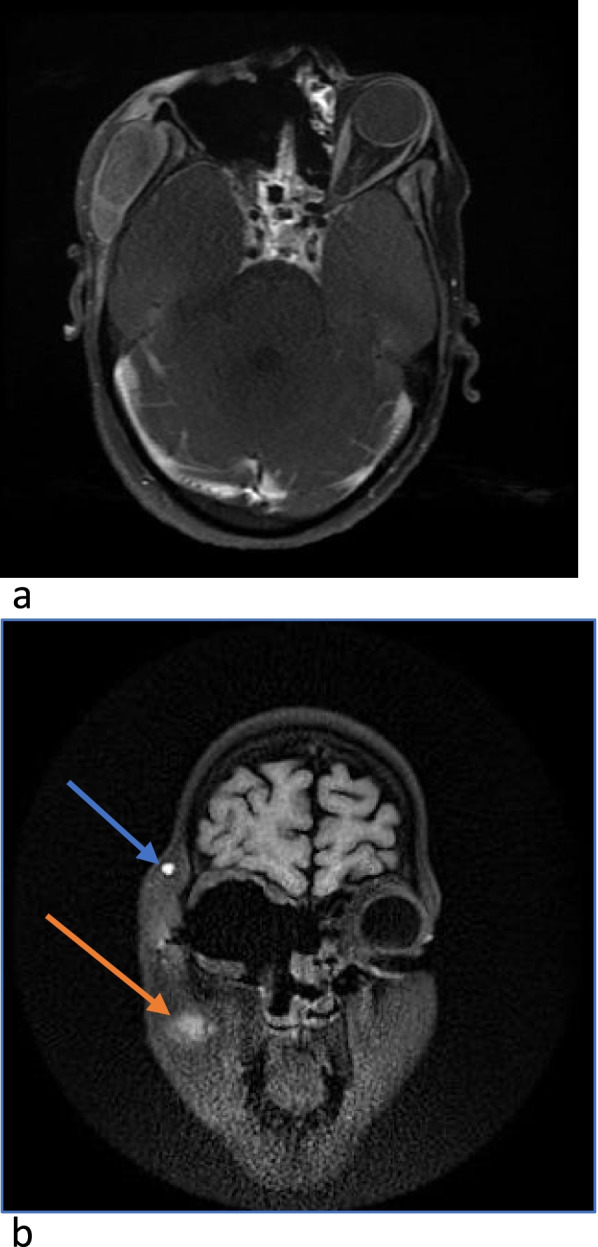
**a**, **b** Post-operative MRI shows right maxillary sinus widening with absent right eye globe and intraocular and extraocular muscles along with removed floor of orbit and absent nasal turbinates on the right side. Right orbit is seen communicating with sphenoid sinus on the right side. Fat suppressed hyperintensity seen in the right temporal fossa(blue arrow) and masticator space(red arrow) involving the right masseter and temporalis muscle. Hyperintensity is seen in the floor of the right maxillary sinus showing peripheral and septal enhancement on post contrast. Bilateral cavernous sinus are patent

The patient had an uneventful postoperative period except for occasional hypokalemia due to amphotericin treatment for which potassium supplementation was given. Her Hb was 9.1 g/dl with a platelet count of 1.38X10^3^/mm3. MRI was done after two weeks of surgery which showed complete recovery and there was no cavernous sinus involvement (Fig. 5a, b).

## Discussion and conclusions

Mucormycosis is a potentially life-threatening condition caused by a group of invasive filamentous fungi of the Mucoraceae family [1, 2]. This fungus is widespread and found in soil, manure, vegetable, fruits, and as bread mold [6]. Most human infections are caused by inhalation of fungal sporangiospores that have been released in the air or by direct inoculation of organisms into disrupted skin or mucosa [7].

Mucormycosis can be predisposed by a history of Covid-19 infection, uncontrolled diabetes, prolonged use of corticosteroids and immunosuppressive drugs, primary and secondary immunodeficiency, haematological malignancies and haematological stem cell transplant, solid organ malignancies and solid organ transplantation, iron overload, etc. [7, 8].

But, in our case patient had developed rhino-orbital mucormycosis with none of these well-known predisposing factors. However, she had a history of P.vivax malaria two weeks prior to the onset of symptoms of rhino-orbital sinusitis which was successfully treated with artemisinin-based combination therapy. Although parasite clearance was not confirmed by laboratory tests post-treatment, peripheral blood smear and rapid diagnostic tests done by us were negative for the malarial parasite. Considering the endemicity of malaria in the Indian subcontinent, this occurrence of mucormycosis following malarial infection can be a coincidental finding. However, cases of invasive mycosis following malaria have been reported, raising concern regarding the possibility of a temporal association between the two.

Plasmodium vivax was long considered a benign infection but has now emerged as a cause of severe and fatal malaria despite its low biomass [9]. They have a greater cytokine release and inflammatory response than plasmodium falciparum malaria. A major distinguishing factor between vivax and falciparum is its low pyrogenic threshold [9]. Severe anemia in vivax is associated with bouts of hemolysis of predominantly uninfected erythrocytes with increased fragility [9]. They show an increased deformity of infected red blood cells and an apparent paucity of parasite sequestration [9].

In a Minireview on the invasive fungal infection (IFI) subsequent to Plasmodium falciparum malaria, Aspergillus species was reported in the majority of the cases [10]. Fungal infection developed or persisted despite clearance of parasitemia. This occurrence of opportunistic fungal infection in falciparum malaria has been attributed to a variety of factors like suppression of innate/adaptive immunity by the organism itself or hemozoin, the release of free iron by hemolysis, and immunosuppression caused by antimalarial treatment [11]. Organs commonly affected by IFI include the lungs, kidney, brain, and heart [12].

Association between IFI and other Plasmodium species causing malaria has been rarely reported. One case of coincidental Plasmodium knowlesi malaria and mucormycosis fungal infection has been reported where the patient had developed lower gastrointestinal bleeding due to mucormycosis, from the twelfth day of antimalarial treatment (after 19 days from onset of fever) [10]. He was successfully treated with 6 weeks of liposomal amphotericin B.

No published data is available regarding the association of P.vivax malaria and mucormycosis. However, it has been shown that P.vivax also causes immunosuppression through dysregulation of neutrophils and dendritic cells [10]. This can also act as a precursor for opportunistic infections.

The first case of rhinomaxillary mucormycosis following severe falciform malaria was reported by Katherine et al. [4]. Symptoms of sinusitis appeared after 16 days of starting artemisinin based therapy(about 5 weeks after onset of fever due to malaria). However, the patient had also received high dexamethasone therapy during treatment for hemolysis. He was managed successfully by nine days of oral itraconazole (until the availability of amphotericin B) and 14 days of deoxycholate amphotericin B. A similar case was reported by Wilson et al. [5]. But, in our case, the duration between the onset of malaria and sinusitis was comparatively shorter (3 weeks). Also, the course of mucor infection was more severe, with the patient requiring surgical management along with antifungal treatment. This can be due to the late initiation of antifungal therapy.

The possibility of underlying thrombocytopenia and anemia leading to mucor infection can also be considered in our case, as hematological disorders can also result in opportunistic mucor infection including profound thrombocytopenia and certain types of anemia like hemolytic or aplastic anemia [13].

This is because platelets apart from their role in hemostasis can also provide a defensive innate response against IFI. This is accomplished by either a direct attack on a fungal pathogen via platelet microbicidal peptides or an indirect phagocytic response by activating monocytes and macrophages [14].

So associated anemia and thrombocytopenia in this patient might also have played a contributory role in this rare occurrence. However, it can also be a coincidental finding as a similar hematological profile is a known complication of P.vivax malaria [1, 3, 7]. Several mechanisms have been proposed for the occurrence of malarial thrombocytopenia including immune complexes mediated peripheral destruction [2, 3, 6] and phagocytosis of platelets leading to low platelet levels. Although thrombocytopenia usually improves within 5–6 days of successful anti-malarial treatment, some studies have reported persistent thrombocytopenia even after complete recovery [15]. Concurrent occurrence of thrombocytopenia can also be explained as a sequela of mucormycosis [1] or a rare adverse effect of rivaroxaban [7].

Several anti-malarial, anticoagulant drugs can also cause drug-induced hemolysis. Delayed hemolysis can also occur 1–3 weeks after initiation of artemisinin-based antimalarial treatment [15]. This can be a possible explanation for anemia as in our case.

Rhino-orbital mucormycosis can pose a diagnostic challenge especially when no predisposing risk factors are present. As there are no hallmark clinical or radiological features, its diagnosis is confirmed only on tissue culture and histopathology. Being a life-threatening condition, prompt antifungal treatment should be started in case of a high index of suspicion, irrespective of the tissue biopsy report.

Since the patient in our case was not improving on broad-spectrum antibiotics, and there was the involvement of all sinuses on the right side, a provisional diagnosis of fungal infection was considered. Nasal examination and biopsy were conducted. Although it was negative for any fungal element, amphotericin B was started. As the patient further deteriorated despite antibiotic and antifungal treatment, surgical debridement of all involved sinus was planned through FESS along with orbital exenteration. Later histopathological examination of exenterated orbital tissue confirmed the diagnosis of mucormycosis and antifungal treatment was continued.

To conclude rhinoorbital mucormycosis can also be seen in absence of predisposing factors. Hence, its possibility should not be ruled out even if such risk factors are absent since mucormycosis can occur in an immunocompetent patient [7].

Antifungal therapy must not be delayed and should be initiated in suspicious cases.

Although is no conclusive evidence regarding the association of mucormycosis with malaria but the possibility of malaria infection predisposing to fungal infection cannot be ruled out. Thus, a population-based study can be done to find out any causal association between the two, especially in malaria-endemic areas.

## Data Availability

All data supporting our findings is contained within the manuscript.

## Abbreviations

LE: Left eye
RE: Right eye
PL: Perception of light
KOH: Potassium hydroxide stain
FESS: Functional endoscopic sinus surgery
IFI: Invasive fungal infection
